# Pinhole does not increase screening accuracy of detecting decreased best corrected visual acuity in schoolchildren

**DOI:** 10.1186/s12886-021-02150-8

**Published:** 2021-12-02

**Authors:** Weiwei Chen, Jing Fu, Ali Sun, Lei Li, Yunyun Sun, Zhaojun Meng

**Affiliations:** Beijing Tongren Eye Center, Beijing Tongren Hospital, Capital Medical University; Beijing Ophthalmology & Visual Sciences Key Laboratory, No.1 Dong Jiao Min Xiang, Beijing, 100730 China

**Keywords:** Sensitivity, Specificity, Decreased best-corrected visual acuity, Vision screening, Schoolchildren

## Abstract

**Background:**

Decreased best corrected visual acuity among children should be treated early in life, and vision screening in schoolchildren is an efficient and feasible selection for developing countries. Thus, the screening accuracy of different visual acuity tests is the key point for making vision screening strategies. The present study aims to explore the screening accuracy of uncorrected visual acuity (UCVA) and pin-hole corrected visual acuity (PCVA) using different vision chart in the detection of decreased best-corrected visual acuity (BCVA) among schoolchildren.

**Methods:**

Grade one primary schoolchildren in urban Lhasa with data of UCVA using tumbling E chart (UCVAE), PCVA using tumbling E chart (PCVAE), UCVA using Lea Symbols chart (UCVAL), PCVA using Lea Symbols chart (PCVAL) and BCVA using Lea Symbols chart were reviewed. Decreased BCVA was defined as BCVA≤20/32(≥0.2 logMAR). Difference, reliability, and diagnostic parameters in the detection of decreased BCVA of different visual acuity results were analyzed.

**Results:**

Overall, 1672 children aged 6.58 ± 0.44 years fulfilling the criteria. The prevalence of decreased BCVA was 6.8%. Although no significant differences were found between UCVAE vs UCVAL (*p* = .84, paired t-test) as well as PCVAE vs PCVAL (*p* = .24), the ICC between them was low (0.68 and 0.57, respectively). The average difference between BCVA and UCVAE, UCVAL, PCVAE, PCVAL was logMAR -0.08 (− 0.37, 0.21), − 0.08 (− 0.29, 0.17), − 0.05 (− 0.30, 0.19), − 0.06 (− 0.23, 0.12) using Bland–Altman method. The area under the receiver operating characteristic curve of UCVAE, PCVAE, UCVAL, PCVAL for the detection of decreased BCVA was 0.78 (0.73, 0.84), 0.76 (0.71, 0.82), 0.95 (0.94, 0.96), 0.93 (0.91, 0.95), respectively.

**Conclusion:**

Pinhole does not increase the screening accuracy of detecting decreased BCVA in grade one primary schoolchildren. Visual acuity test using Lea Symbols is more efficient than Tumbling E in the screening of that age.

**Trial registration:**

Data were maily from the Lhasa Childhood Eye Study which has finished the clinical registration on (ChiCTR1900026693).

## Background

Decreased best corrected visual acuity (BCVA) such as amblyopia is a common vision disorder among children. The prevalence of amblyopia in preschool and school populations are ranging from 0.18 to 4.70% [1–3]. Most of the conditions of decreased BCVA can be improved or eliminated more easily when treated early in life. Left untreated, vision abnormalities in young children could lead to permanent loss of vision, as well as problems at school, bullying, reduced functionand quality of life, depression, anxiety, and injuries [4]. Untreated amblyopia rarely resolves spontaneously [5].

The US Preventive Services Task Force recommended children aged 3 to 5 years to detect amblyopia or its risk factors [2, 5]. It is difficult for developing countries to perform vision screening in pre-school children as it’s costly and labor-intensive. Vision screening in schoolchildren is more efficient and feasible. It is suggested that visual acuity assessment in children over 3 years old is an accurate method to detect amblyopia and its risk factors. Different visual acuity chart has its advantages, disadvantages, and applicable age. Many studies established that Lea’s visual acuity has better sensitivity in preschool vision screening [6–10]. As Lea Symbols visual acuity chart was expensive and not easy to buy in China, it was worth to evaluate the advantages and disadvantages in large-scale vision screening in schoolchildren. Although uncorrected visual acuity (UCVA) is sensitive to detect amblyopia, the specificity is low. There is adequate evidence in many epidemiology studies that 47–92.7% of the reduced vision in school-age children is caused by uncorrected refractive error [11]. The World Health Organization-endorsed rapid assessment of avoidable blindness survey employs pinhole acuity to distinguish between refractive error versus conditions not correctable with eyeglasses [12]. Pin-hole corrected visual acuity (PCVA) is a potential method that is easy to perform in vision screening to improve the accuracy of decreased BCVA detection [13]. But it is important to pursue whether it is necessary to perform considering the cost and manpower needed in vision screening, particularly in developing countries.

The purpose of the present study is to explore the screening accuracy of UCVA and PCVA using tumbling E or Lea Symbols chart in the detection of decreased BCVA among schoolchildren in urban Lhasa using data of the Lhasa Childhood vision Screening (LCVS) and the Lhasa Childhood Eye Study (LCES) [14].

## Methods

This diagnostic study was based on the data from a mandatory vision screening named LCVS conducted in urban Lhasa and the baseline data of a school-based childhood cohort study named LCES. The study adheres to the principles of the Declaration of Helsinki. No individual-participant data were used. The LCVS did not require parental consent since the study involved no intervention beyond screening, and school principals were children’s legal guardians in China. LCES has finished the clinical registration on http://www.chictr.org.cn/showprojen.aspx?proj=44165 (ChiCTR1900026693). Ethics committee approval was obtained from the Institutional Review Board of Beijing Tongren Hospital, Capital Medical University (TRECKY2019–146). Informed consent forms signed by the parents of all the participants were obtained before the start of the LCES.

### Study area

LCVS and LCES were carried out among urban primary school children in Lhasa. Lhasa is located in the middle of the Tibetan plateau, China with an average altitude of 3650 m. The total population is 90.25 million, mainly of which is Tibetan. Lhasa has three districts and five counties. The enrollment rate of primary school-age children is 99.7%. The three urban districts of Lhasa selected for LCVS and LCES have 28 elementary schools with approximately 40,000 primary school students. Primary education in Lhasa lasts for 6 years.

### Visual acuity assessment in LCES

Grade one students from primary schools in Lhasa were cluster randomly selected. They were examined annually for 5 years. The examination procedures for LCES consisted of standardized ocular, systematic examinations, and questionnaires. Standardized ocular examinations included distant, near and pin-hole visual acuity, identification of amblyopia and strabismus, ocular biometry, optical coherence tomography, retinal photography, cycloplegic autorefraction, intraocular pressure, stereo acuity, and ocular dominance. Objective refraction was measured before and after cycloplegia using an autorefractor (KR-800, Topcon, Tokyo, Japan) followed by subjective refraction by trained optometrists. Each student was first administered one drop of topical anesthetic agent (Alcaine, Alcon) to alleviate discomfort, followed by two drops of 1% cyclopentolate (Alcon) and 1 drop of Mydrin P (Santen, Japan) after a 5-min interval. 30 min after the last drop, thethird drop of cyclopentolate would be administered if pupillary light reflex will be still present or the pupil size will be less than 6.0 mm. The baseline data collection was conducted from October to November 2019. Detailed information of LCES could be found in our published paper [14]. UCVA was measured for the right eye and left eye using Lea Symbols ETDRS 3 m Set charts (250,300, Goodlite, IL, USA) at a distance of 3 m. On each measurement, the contralateral eye was occluded and the subject is asked to read the figure on the right edge of each line (starting from the top line) until they made a mistake, then the subject’s attention was drawn to the 2 lines above the line on which they made their initial mistake. The subject was asked to read each figure on successive lines until they made 3 or more mistakes on a line. The last line attempted, combined with the number of mistakes made on that and previous lines, was used to calculate a letter-by-letter logMAR visual acuity score. PCVA and best-corrected distant visual acuity would be obtained after a subjective refraction test for students with UCVA < 20/20.

### Visual acuity assessment in LCVS

LCVS was led by the Lhasa municipal government. The screening protocol was designed by the Strabismus and Pediatric Ophthalmology Department of Beijing Tongren Hospital and conducted by trained volunteers with the permission of all the principals of the participating children. The screening was conducted from July 1 to September 20 of 2019. UCVA was examined monocularly for all the participants through tumbling E Standard Logarithm Eyesight Table at a distance of 5 m. Visual acuity was assessed monocularly with the right eye tested first. The left eye was tested first only in previously diagnosed cases of amblyopia in the left. An opaque, black occluder was used to cover the eye not being tested during visual acuity assessment. The threshold visual acuity testing was used in LCVS. The examiner asked the child to start at the top of the eye chart and continue reading down each line until the child recited the smallest line of optotypes discernable. Visual acuity was noted as the finest line, where over half of the optotypes were recognized. Students with UCVA of equal to or more than 20/25 in both eyes passed the screening. For students with UCVA poorer than 20/25 in either eye, a retest of pin-hole corrected distant visual acuity was obtained. Vision values were converted to a logarithm of minimal angle of resolution (logMAR) units for further statistical analysis.

### Inclusion and exclusion criteria for participants of the present study

All the primary school students in urban Lhasa were encouraged to participate in LCVS except the principals or the parents of the students refused and addressed the reasons to the local government. Individuals suffering from mental illness or other medical conditions that are unable to cooperate with the test were excluded. Voluntary grade one students, living in Lhasa city for at least half a year and planning to continue to live there for at least 5 years, are legible for LCES. Individuals, suffering from mental illness or other medical conditions, were unable to cooperate with the baseline survey and follow-up would be excluded. All the students with data of uncorrected distant visual acuity using tumbling E (UCVAE), pin-hole corrected visual acuity using tumbling E (PCVAE), UCVAL (UCVAL), pin-hole corrected visual acuity using Lea Symbols chart (PCVAL), and BCVA were selected in the present study. All the analyzations were based on the data from the right eyes.

### Data processing and diagnosis of decreased BCVA

All the data from LCVS and LCES was filled in paper forms and were independently entered into the database using Epidata software 3.1 (The Epidata Association, Odense, Denmark) by two individuals separately. Data cleaning of each study was done within 1 month after the data collection. Data merging of LCVS and LCES was conducted. Right eyes with data of UCVA and PCVA using both tumbling E and Lea Symbols chart as well as best-corrected distant visual acuity were selected for further analysis. The PCVA was recorded as UCVA when UCVA≥20/25. The best-corrected distant visual acuity was recorded as PCVA when PCVAL≥20/20. Decreased BCVA was defined as best-corrected distant visual acuity≤20/32(≥0.2 logMAR) based on the baseline data of LCES.

### Statistical analysis

Statistical analysis was performed using SAS software (version 9.4, SAS Inc., Cary, NC, USA). The characteristics of the research subjects were summarized with means ± standard deviation, frequencies, and percentages when appropriate. Paired t-test was used to analyze the values of different visual acuity Tests. The reliability between different results of visual acuity was assessed with the intraclass correlation coefficient and Bland–Altman plots. Receiver operating characteristic curves were used to determine the optimal referral cutoff values for each test.

## Results

### Characteristics of the participants

**Fig. 1:**
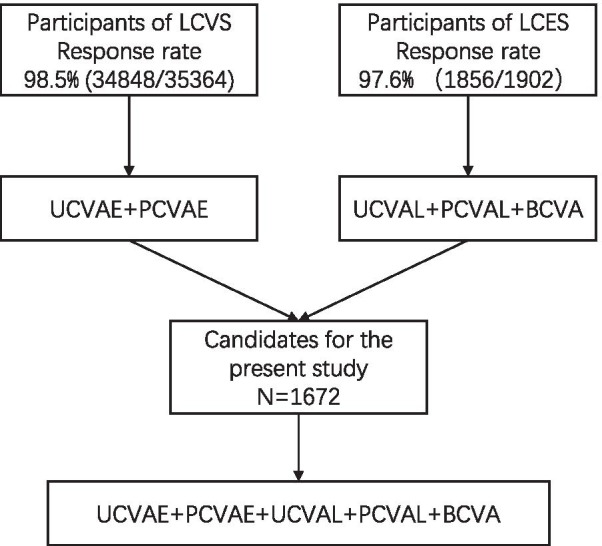
Recruitment of the candidates for the present study. LCVS, Lhasa Childhood Vision Screening; LCES, Lhasa Childhood Eye Study; UCVA, uncorrected visual acuity; PCVA, pin-hole corrected visual acuity; BCVA, best corrected visual acuity; UCVAE, uncorrected visual acuity using tumbling E chart; PCVAE, pin-hole corrected visual acuity using tumbling E chart; UCVAL, uncorrected visual acuity using Lea Symbols char; PCVAL, pin-hole corrected visual acuity using Lea Symbols chart

The response rate of LCVS and LCES was 98.5% (34,848/35364) and 97.6% (1856/1902), respectively. There were 1672 grade one students fulfilling the criteria for the present study. Recruitment details were shown in Fig. 1. The mean age of the participants was 6.58 ± 0.44 years, 52.2% (873/1672) were males. 94.9% (1587/1672) of the participants were Tibetan. The prevalence of decreased best-corrected distant visual acuity was 6.8%. Overall participant characteristics were shown in Table 1.

**Table 1:** Characteristics of participants in the present study (*n* = 1672)

Characteristics	n (%)	mean ± SD
Age, years		6.58 ± 0.44
Gender, male	873(52.2)	
Ethnic categories		
Tibetan	1587(94.9)	
Han	77(4.6)	
Others	8(0.5)	
Amblyopia	114(6.8)	
UCVAE		0.10 ± 0.17
PCVAE		0.08 ± 0.13
UCVAL		0.10 ± 0.16
PCVAL		0.08 ± 0.13
BCVA		0.03 ± 0.10

Data presented are mean ± SD or frequency (%), where appropriate. All visual acuities were recorded as logMAR visual acuity score. *LCVS* Lhasa Childhood Vision Screening; *UCVA* uncorrected visual acuity; *PCVA* pin-hole corrected visual acuity; *BCVA* best-corrected visual acuity; *UCVAE* UCVA using tumbling E chart; *PCVAE* PCVA using tumbling E chart; *UCVAL* UCVA using Lea Symbols char; *PCVAL* PCVA using Lea Symbols chart

### Comparison and correlations between results of different visual acuity tests

**Fig. 2:**
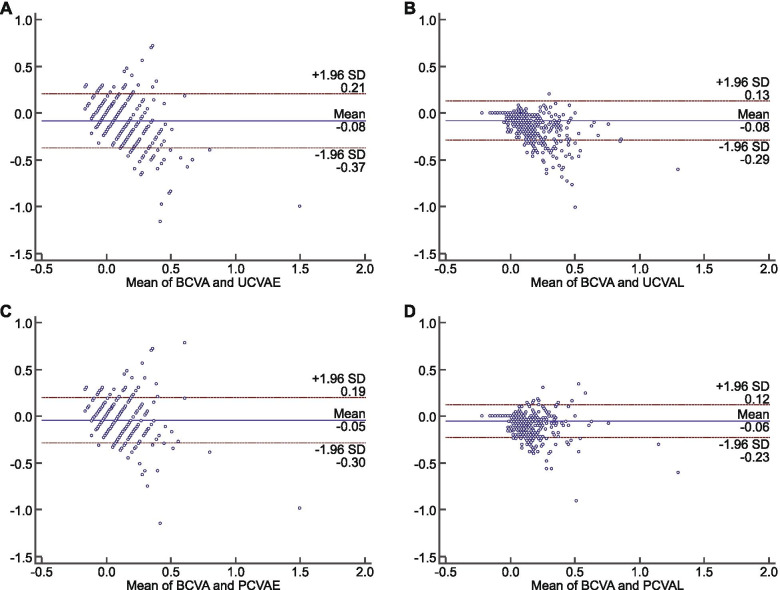
Bland–Altman plots for the results of different Visual Acuity Tests. BCVA, best corrected visual acuity; UCVAE, uncorrected visual acuity using tumbling E chart; PCVAE, pin-hole corrected visual acuity using tumbling E chart; UCVAL, uncorrected visual acuity using Lea Symbols char; PCVAL, pin-hole corrected visual acuity using Lea Symbols chart. **A**, Bland–Altman plot of BCVA vs UCVAE; **B**, plot of BCVA vs UCVAL; **C**, plot of BCVA vs PCVAE; **D**, plot of BCVA vs PCVAL. The central line represents the absolute average difference between instruments, while the upper and the lower lines represent ±1.96 standard deviation. BCVA, best corrected visual acuity; UCVAE, UCVA using tumbling E chart; PCVAE, PCVA using tumbling E chart; UCVAL, UCVA using Lea Symbols char; PCVAL, PCVA using Lea Symbols chart

The comparison and intraclass correlation coefficient between different visual acuity results were list in Table 2. Although no significant differences were found between UCVAE vs UCVAL (*p* = .84) as well as PCVAE vs PCVAL (*p* = .24), the intraclass correlation coefficient between them was low (0.68 and 0.57, respectively). A higher intraclass correlation coefficient was found between best-corrected distant visual acuity vs PCVAE (0.84). On average, all the visual acuity results measured were less than a line different from best-corrected distant visual acuity. Bland–Altman results were shown in Fig. 2. The average difference between best-corrected distant visual acuity and UCVAE, UCVAL, PCVAE, PCVAL was logMAR -0.08 (95% CI: − 0.37, 0.21), − 0.08 (95% CI: − 0.29, 0.17), − 0.05 (95% CI: − 0.30, 0.19), − 0.06 (95% CI: − 0.23, 0.12).

**Table 2:** The ICC between different VA results

Parameters	T	P^a^	ICC(95%CI)	P^b^
UCVAE vs UCVAL	0.20	0.84	0.68(0.64, 0.71)	< 0.01
PCVAE vs PCVAL	1.19	0.24	0.57(0.52, 0.61)	< 0.01
BCVA vs UCVAE	21.68	< 0.01	0.59(0.54, 0.62)	< 0.01
BCVA vs PCVAE	16.95	< 0.01	0.57(0.53, 0.61)	< 0.01
BCVA vs UCVAL	29.72	< 0.01	0.81(0.79, 0.83)	< 0.01
BCVA vs PCVAE	25.86	< 0.01	0.84(0.82, 0.85)	< 0.01

a, *P* value of pared t-test; b, P value of intraclass correlation coefficient. *ICC* intraclass correlation coefficient; *VA* visual acuity; *95%CI* 95% confidence interval; *UCVA* uncorrected visual acuity; *PCVA* pin-hole corrected visual acuity; *BCVA* best corrected visual acuity; *UCVAE* UCVA using tumbling E chart; *PCVAE* PCVA using tumbling E chart; *UCVAL* UCVA using Lea Symbols char; *PCVAL* PCVA using Lea Symbols chart

### Receiver operating characteristic curve analysis

**Fig. 3:**
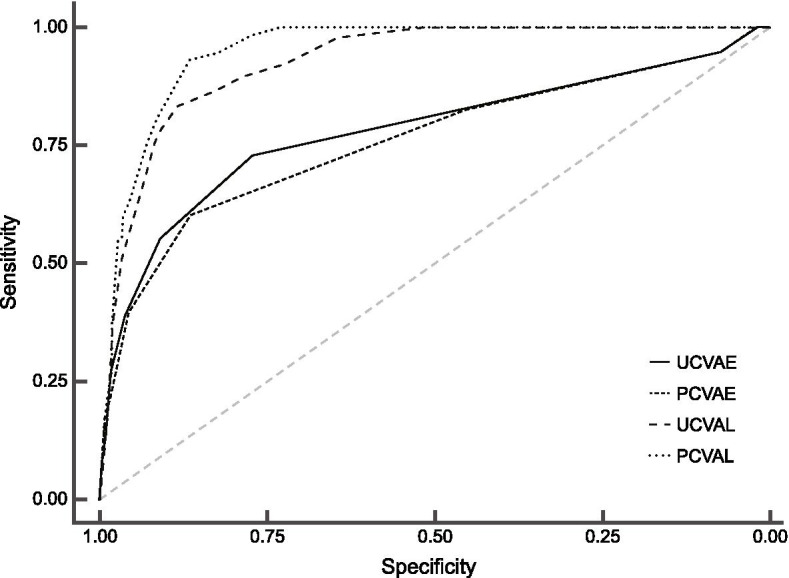
Receiver operating characteristic curves for the detection of decreased best corrected visual acuity. ROC, receiver operating characteristic; UCVAE, uncorrected visual acuity using tumbling E chart; PCVAE, pin-hole corrected visual acuity using tumbling E chart; UCVAL, uncorrected visual acuity using Lea Symbols char; PCVAL, pin-hole corrected visual acuity using Lea Symbols chart. The area under the ROC curve (AUC) of UCVAE, PCVAE, UCVAL, PCVAL for the detection of decreased best corrected visual acuity was 0.78, 0.76, 0.95, 0.93, respectively

The receiver operating characteristic curves of the different visual acuity measurements for detecting the decreased best-corrected distant visual acuity were shown in Fig. 3.The area under the receiver operating characteristic curve of UCVAE,PCVAE, UCVAL, PCVAL for the detection of decreased best-corrected distant visual acuity was 0.78 (95%CI: 0.73, 0.84), 0.76 (0.71, 0.82), 0.95 (0.94, 0.96), 0.93 (0.91, 0.95), respectively. The visual acuity cutoffs with best discriminative capacity and their sensitivity, specificity, positive predictive value, and negative predictive value were shown in Table 3.

**Table 3:** AUC, best cut off, and its diagnostic values for each VA measurement

	AUC(95%CI)	p	Best cut-off	sensitivity	specificity	PPV	NPV
UCVAE	0.78 (0.73, 0.84)	< 0.001	0.16	0.73	0.77	0.18	0.97
PCVAE	0.76 (0.71, 0.82)	< 0.001	0.16	0.61	0.86	0.25	0.97
UCVAL	0.95 (0.94, 0.96)	< 0.001	0.19	0.93	0.87	0.34	0.99
PCVAL	0.93 (0.91, 0.95)	< 0.001	0.19	0.83	0.88	0.34	0.99

*AUC* the area under the ROC curve; *PPV* positive predictive value; *NPV* negative predictive value; *UCVA* uncorrected visual acuity; *PCVA* pin-hole corrected visual acuity; *UCVAE* UCVA using tumbling E chart; *PCVAE* PCVA using tumbling E chart; *UCVAL* UCVA using Lea Symbols char; *PCVAL* PCVA using Lea Symbols chart

## Discussion

The present study reviewed 1672 records for a school-based preschool eye study and a population based vision-screening program. The adequate sample size is important to ensure the power in a confirmatory diagnostic accuracy study [15]. Assuming a sensitivity of 0.8, a tolerated error of 0.1, a level test of 0.05, and the prevalence of decreased best-corrected distant visual acuity of 6.8%, the minimum sample size would be 904 [16]. When calculating using specificity of 0.8, the minimum sample size would be 66 [16]. The effectiveness of this study is sufficient. Vision screenings are important in the goal of long term reduction of vision loss in childhood. In a joint position statement, vision screening was recommended for school-aged children every 1–2 years [4, 5, 17]. The US Preventive Services Task Force stated that there was adequate evidence that vision screening in children aged 3 to 5 yearsto detect amblyopia or its risk factorsimproved visual acuity [5]. However, it is difficult for most developing countries to perform vision screening in preschool children. In some regions of China, children received the first ophthalmic test in primary school. Even in the US, less than 22% of preschool children receive some type of vision screening according to the Centers for Disease Control and Prevention [18].It was meaningful to evaluate the accuracy of the commonly used visual acuity screening test.

Best-corrected distant visual acuity is a measure of the smallest level of detail that can be resolved in an image, typically measured on a chart with letters or pictures of reducing the size (optotypes) while wearing full spectacle correction [19]. We defined decreased best-corrected distant visual acuity as best-corrected distant visual acuity≤20/32(≥0.2 logMAR) using Lea symbol chart in the present study. Because bilateral amblyopia is defined as a reduction of 0.20 logMAR or more compared with the developmental norms for best-corrected distant visual acuity at a given age clinically. Grade one primary school students who are averaged 6-year old are expected to have a best-corrected distant visual acuity of 0.00 logMAR [20]. The prevalence of decreased best-corrected distant visual acuity was 6.8%. This was higher than the prevalence of amblyopia (raged from 1 to 5%) reported in many epidemiology studies [21–23]. We suspected it was mostly because students with decreased best-corrected distant visual acuity included other ophthalmic abnormal.

The present results demonstrated that visual acuity using Lea Symbols was more efficient than Tumbling E in screening for detection of decreased best-corrected distant visual acuity in grade one primary schoolchildren. Although no significant difference was found in the comparison of the means between UCVAE vs UCVAL as well as PCVAE vs PCVAL, the intraclass correlation coefficient between them was low (0.68 and 0.57, respectively). The area under the receiver operating characteristic curve of visual acuity using tumbling E was less than 0.8, while the area under the receiver operating characteristic curve of visual acuity using Lea symbol was more than 0.9. One possibility is that the record of the tumbling E chart in this study is a threshold of a whole line whereas the record of Lea symbol chart is able to distinguish every letter recognized. The best-corrected distant visual acuity was tested with Lea Symbol chart. This might influence the diagnostic results, too. Directional optotypes as tumbling E charts are widely used in non-spoken English countries. The procedure of a vision test using tumbling E in the present study was nationally suggested in China. This condition made the price of the vision chart using tumbling E much lower than Lea symbol chart (45 vs 420 dollars). Considering the lower screening accuracy of the tumbling E chart, a new expert’s advice and a vision chart production change were needed.

This study also showed that pinhole did not increase the screening accuracy of detecting decreased best-corrected distant visual acuity in grade one primary schoolchildren. UCVA is probably the best single-instrument test in developing countries or areas with low resources, especially for children aged 5 years or more [20, 22]. The World Health Organization endorsed rapid assessment of avoidable blindness (RAAB) survey employs pinhole acuity to distinguish between refractive error versus conditions not correctable with eyeglasses in the adult [24]. A series of 16 RAABs included a sampling of children < 15 years were conducted in Vietnam and these to allow estimation of the prevalence and causes of blindness in children [25].We found that the areas under the receiver operating characteristic curve ofPCVAE (0.76) and PCVAL (0.91) were not higher than UCVAE (0.78) and UCVAL (0.95) for the detection of decreased best-corrected distant visual acuity. We hypothesized that this might be due to the relatively lower prevalence of refractive error in children of this age. We found that the average difference between best-corrected distant visual acuity and PCVAE/PCVAL was less than 3 letters. This was similar to the results of Rajesh et al. [ 11] The results gave evidence that there was no need to add PCVA at least in grade one primary schoolchildren because of the added burden of cost and manpower in developing countries. We found that it took more than double the time to screen a child when adding PCVA. As the screening samples increased, the manpower and economic costs would increase. Further health economics evaluation studies were needed to address the extra costs and the necessity of adding PCVA in the vision screening in schoolchidren of different grades.

The major achievement of our study is that this is a school-based diagnostic study with a large sample size, guarantying the evaluation power. We have verified the screening accuracy by using various test combinations and the receiver operating characteristic curve, which would help institutes conducting vision screening programs in schoolchildren. Nevertheless, a limitation of the study is the response rate as a part of the cohort study LCES is relatively lower (87.9%, 1672/1902) resulting from the missing data of visual acuity using the tumbling E chart performed in the vision screening 1 month before LCES. However, as discussed above the sample size is adequate for a confirmatory diagnostic accuracy study. Besides, only grade one students are involved in the program. Following-up data of LCES will make up the defect.

In conclusion, these data of vision screening indicate visual acuity test using Lea Symbols is more efficient than Tumbling E in the screening of grade one primary schoolchildren. It also suggests that pinhole visual acuity does not increase the screening accuracy of detecting decreased best-corrected distant visual acuity in children of that age. Our results potentially provide evidencethat the Lea Symbol visual acuity chart is worthy to be promoted and the pin hole visual acuity can be canceled in large scale vision screening programs of grade one primary schoolchildren in developing countries.

## Data Availability

The datasets used and/or analyzed during the current study are available from the corresponding author on reasonable request.

## Abbreviations

LCES: Lhasa Childhood Eye Study
LCVS: Lhasa Childhood Vision Screening
UCVA: Uncorrected visual acuity
PCVA: Pin-hole corrected visual acuity
BCVA: Best corrected visual acuity
UCVAE: Uncorrected visual acuity using tumbling E chart
PCVAE: Pin-hole corrected visual acuity using tumbling E chart
UCVAL: Uncorrected distant visual acuity using Lea Symbols chart
PCVAL: Pin-hole corrected visual acuity using Lea Symbols chart

## References

[1] Fu Z, Hong H, Su Z (2020). Global prevalence of amblyopia and disease burden projections through 2040: a systematic review and Meta-analysis. Br J Ophthalmol.

[2] Jin J (2017). Vision screening in children. JAMA.

[3] Hunter D, Cotter S (2018). Early diagnosis of amblyopia. Vis Neurosci.

[4] Silverstein E, Donahue SP (2018). Preschool Vision Screening: Where We Have Been and Where We Are Going. Am J Ophthalmol.

[5] Jonas DE, Amick HR, Wallace IF (2017). Vision screening in children aged 6 months to 5 years: evidence report and systematic review for the us preventive services task force. JAMA.

[6] Sanker N, Dhirani S, Bhakat P. Comparison of visual acuity results in preschool children with lea symbols and bailey-lovie e chart. Middle East Afr J Ophthalmol2013;20:345–348. 10.4103/0974-9233.120020 PMC3841955 24339687

[7] Paul C (2018). M; Sathyan, Sanitha. Comparison of the efficacy of Lea symbol chart and Sheridan Gardiner chart for preschool vision screening. Indian J Ophthalmol.

[8] Gräf MH, Becker R, Kaufmann H (2000). Lea symbols: visual acuity assessment and detection of amblyopia. Graefes Arch Clin Exp Ophthalmol.

[9] Moganeswari D, Thomas J, Srinivasan K (2015). Test re-test reliability and validity of different visual acuity and Stereoacuity charts used in preschool children. J Clin Diagn Res.

[10] Thomas J, Rajashekar B, Kamath A (2020). Diagnostic accuracy and agreement between visual acuity charts for detecting significant refractive errors in preschoolers. Clin Exp Optom..

[11] Atowa UC, Hansraj R, Wajuihian SO. Visual problems: a review of prevalence studies on visual impairment in school-age children. Int J Ophthalmol2019;12:1037–1043. 10.18240/ijo.2019.06.25 PMC6580204 31236365

[12] K Kumar RS, Rackenchath MV, Sathidevi AV, et al. (2019). Accuracy of pinhole visual acuity at an urban Indian hospital. Eye (Lond).

[13] Marmamula S, Keeffe JE, Narsaiah S (2014). Population-based assessment of sensitivity and specificity of a pinhole for detection of significant refractive errors in the community. Clin Exp Optom.

[14] Chen W, Fu J, Meng Z (2020). Lhasa childhood eye study: the rationale, methodology, and baseline data of a 5 year follow-up of school-based cohort study in the Tibetan plateau region of Southwest China. BMC Ophthalmol.

[15] Stark M, Zapf A (2020). Sample size calculation and re-estimation based on the prevalence in a single-arm confirmatory diagnostic accuracy study. Stat Methods Med Res.

[16] Yao X, Vella E (2017). How to conduct a high-quality original study on a diagnostic research topic. Surg Oncol.

[17] Solebo AL, Cumberland PM, Rahi JS (2015). Whole-population vision screening in children aged 4-5 years to detect amblyopia. Lancet.

[18] Shakarchi AF, Collins ME (2019). Referral to Community Care from School-Based Eye Care Programs in the United States. Surv Ophthalmol.

[19] Pilling RF, Outhwaite L (2017). Are all children with visual impairment known to the eye clinic?. Br J Ophthalmol.

[20] Tailor V, Bossi M, Greenwood JA, Dahlmann-Noor A (2016). Childhood amblyopia: current management and new trends. Br Med Bull.

[21] Margines JB, Huang C, Young A (2020). Refractive errors and amblyopia among children screened by the Ucla preschool vision program in Los Angeles County. Am J Ophthalmol.

[22] Zhao L, Stinnett SS, Prakalapakorn SG (2019). Visual acuity assessment and vision screening using a novel smartphone application. J Pediatr.

[23] Good WV (2017). Vision screening in very Young children-making sense of an inexorable diagnostic process. JAMA Pediatr.

[24] Mactaggart I, Limburg H, Bastawrous A (2019). Rapid assessment of avoidable blindness: looking Back, looking forward. Br J Ophthalmol.

[25] Marmamula S, Madala SR, Rao GN (2011). Rapid assessment of visual impairment (Ravi) in marine fishing communities in South India--study protocol and Main findings. BMC Ophthalmol.

